# Testosterone Therapy for the Treatment of Age-Related Hypogonadism: Risks with Uncertain Benefits

**DOI:** 10.1089/andro.2020.0018

**Published:** 2021-05-06

**Authors:** Christine P. Nguyen, Mark Hirsch, Suresh Kaul, Corinne Woods, Hylton V. Joffe

**Affiliations:** ^1^ Division of Urology, Obstetrics and Gynecology, Office of New Drugs, Pediatrics, Urologic and Reproductive Medicine, Office of New Drugs, Center for Drug Evaluation and Research, U.S. Food and Drug Administration, Silver Spring, Maryland, USA.; ^2^ Division of Epidemiology II, Office of Surveillance and Epidemiology, Pediatrics, Urologic and Reproductive Medicine, Office of New Drugs, Center for Drug Evaluation and Research, U.S. Food and Drug Administration, Silver Spring, Maryland, USA.; ^3^ Office of Rare Diseases, Pediatrics, Urologic and Reproductive Medicine, Office of New Drugs, Center for Drug Evaluation and Research, U.S. Food and Drug Administration, Silver Spring, Maryland, USA.

**Keywords:** testosterone, hypogonadism, age-related

## Abstract

Testosterone replacement therapy has been approved in the United States since the 1950s for men with “classical” hypogonadism. These men have specific and well-recognized hypothalamic, pituitary, or testicular conditions leading to deficient or absent endogenous testosterone. A more controversial treatment population is aging men, many with comorbidities, who have low serum testosterone concentrations compared with young healthy men and who do not have the well-recognized medical conditions that cause “classical” hypogonadism. Testosterone continues to be widely used in these men with “age-related hypogonadism” even though the benefits of testosterone for this use are uncertain and there are important risks, including a potential risk of major adverse cardiac events for the testosterone class, and two testosterone products with increases in blood pressure that can increase the risk of myocardial infarction and stroke. Given the uncertain clinical benefit of testosterone in men with “age-related hypogonadism” in the face of known and potential adverse outcomes, none of the testosterone products is FDA approved for such use.

Different formulations and routes of administration for testosterone have been approved by the FDA over the past several decades. The FDA-approved use is for testosterone replacement therapy in men for conditions associated with a deficiency or absence of endogenous testosterone.^1^ These conditions include primary hypogonadism (e.g., testicular failure due to bilateral torsion, orchitis, Klinefelter syndrome, or chemotherapy) as well as hypogonadotropic hypogonadism (e.g., resulting from a pituitary gland tumor or radiation). To support this approval, the clinical development program for a testosterone product only needs to show that the product can reliably restore low serum testosterone concentrations into the eugonadal range for young healthy men.^1^

This approach for “classical” hypogonadism follows the endocrinological paradigm of hormonal replacement for conditions that directly cause the absent or low concentrations of the affected hormone. That is, treatment is the restoration of the low or absent hormone, and it follows that such restoration corrects the clinical consequences of the missing hormone. Therefore, these programs are not designed to show benefit on any sign or symptom of hypogonadism. Instead, for men with “classical” hypogonadism—those with low testosterone concentrations due to specific well-recognized medical conditions such as those already described—the increase in serum testosterone concentration establishes clinical benefit. We know, for example, that a man who develops low testosterone concentrations due to testicular failure resulting from specific etiologies such as those already described would otherwise not have had low testosterone concentrations and that restoring testosterone concentrations in such men is clearly appropriate. The trials conducted for approval of a new testosterone replacement therapy also establish the safety of the product, generally providing data on patients treated for at least 1 year.

In contrast to the approved use, since the early 2000s there has been a large increase in the use of testosterone in men with “age-related hypogonadism.”^1^ These older men have serum testosterone concentrations below the normal range for young healthy men without having any of the specific well-recognized medical conditions that cause “classical” hypogonadism. Although these men may also have signs and symptoms consistent with hypogonadism, it is unclear whether these nonspecific findings (e.g., fatigue, sexual dysfunction) are related to the testosterone concentrations or are caused by coexisting conditions, concomitant medications, or possibly aging itself. These issues raise important questions such as whether “low” serum testosterone concentrations in older men compared with younger men are physiological or pathological, and whether raising serum testosterone concentrations in older men leads to clinical benefits (e.g., improvements in how these men feel, function, or survive).

In 2004, the Institute of Medicine (now the National Academy of Medicine) concluded that the available evidence at that time on the effects of testosterone therapy in older men was limited and inconclusive.^2^ Despite these uncertainties, older men were targeted for many years by direct-to-consumer advertisements for testosterone products and “low T” disease-awareness campaigns that implied unsubstantiated treatment benefits. From 2010 to 2013, ∼80% of prescription testosterone users were men between 40 and 74 years of age with the pattern of use consistent with treatment of “age-related hypogonadism.”^3^ In addition, in a health claims database, 28% of men who received a new testosterone prescription between 2008 and 2013 had no evidence of a prior serum testosterone concentration measurement, a concerning finding given that a key diagnostic criterion for male hypogonadism is a repeated serum testosterone concentration below the eugonadal range.^1^

This significant increase in testosterone use for age-related hypogonadism, despite lack of definitive evidence of benefit together with some studies suggesting potential cardiovascular risk associated with testosterone use, prompted FDA to convene a public advisory committee meeting in 2014 to discuss these issues.^1^ The committee members concluded that the evidence at that time only supported an FDA-approved use in men with “classical” hypogonadism and that there was a signal of cardiovascular risk associated with testosterone therapy.^1^ Based on these recommendations, FDA updated the labeling for testosterone products to state that effectiveness and safety of testosterone have not been established for “age-related hypogonadism,” removed the term “idiopathic” from the Indication and Usage section because this term could be misinterpreted as an approved use for hypogonadism of unclear etiology, and included a warning about the potential for cardiovascular risk with testosterone use. Given the conflicting data and limitations with the existing studies, the FDA also required companies that own testosterone products to conduct a controlled clinical trial to determine the effects of testosterone therapy on major adverse cardiac events (MACE), including cardiovascular death, myocardial infarction, and stroke, and encouraged the companies to work together on a single trial. This trial is underway with an estimated completion date of June 2022, randomizing 6000 symptomatic hypogonadal men 45–80 years old and at increased risk for cardiovascular disease to testosterone gel or placebo gel with a primary end-point of time to MACE (ClinicalTrials.gov Identifier: NCT03518034).

Recently, we evaluated the use of testosterone in the United States from 2014 to 2018 to assess for changes in utilization patterns after the 2014 advisory committee meeting and our revisions to the testosterone labeling. Estimated sales of testosterone products from all manufacturers to all settings of care decreased from ∼54 million packages^i^ in 2014 to 40 million packages in 2016, then steadily increased to 47 million packages in 2019. The estimated number of male patients who received testosterone prescriptions from U.S. outpatient retail pharmacies decreased 27% from an apparent peak of 1.8 million patients in 2013 to 1.3 million patients in 2018 (Fig. 1).^4^ According to U.S. office-based physician surveys, “replace testosterone” was the top diagnosis associated with testosterone in 2016–2017.^5^ In 2017, 21% of patients starting testosterone therapy did not have a claim for a testosterone blood laboratory test before initiation of testosterone therapy, an improvement from the prior 28% finding but still concerning as these data suggest that potentially as many as one in five patients are still being prescribed testosterone without laboratory confirmation of low serum testosterone concentrations.^6^ In summary, there appears to be a decline in testosterone use after the FDA advisory committee meeting and labeling revisions, though testosterone use remains considerable.

**FIG. 1.**
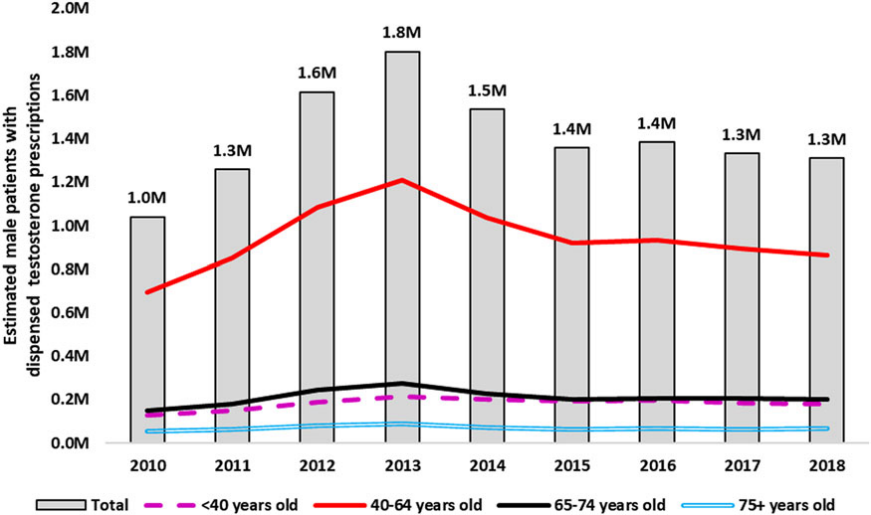
Nationally estimated number of male patients (in millions) with testosterone prescriptions dispensed from U.S. outpatient retail pharmacies, 2010–2018 annually. Source: IQVIA Total Patient Tracker™, 2010–2018, data extracted October 2020.

Is there any new information since our 2015 regulatory actions on testosterone therapies that should alter the conclusions reached at that time? Although there continue to be publications evaluating the cardiovascular safety of testosterone therapy, this remains a controversial issue while we await the results of the required ongoing double-blind placebo-controlled cardiovascular outcomes trial. We have also learned of a new safety concern during the drug development programs for two recently approved testosterone products, subcutaneously administered testosterone enanthate and orally administered testosterone undecanoate.^7^ These products increase systolic blood pressure, on average, by ∼4 mmHg on 24-hour ambulatory blood pressure monitoring, a sensitive monitoring technique that was not routinely used in previous testosterone product development programs. In some patients, the increase in blood pressure may be too small to detect in clinical practice with routine cuff measurements and would not necessarily lead to initiation or changes in blood pressure medications. However, these blood pressure increases have been well established with longitudinal data to increase the risk for MACE over time, including the risks of cardiovascular death, myocardial infarction, and stroke, with greater risk seen in patients with established cardiovascular disease or risk factors for cardiovascular disease (which is anticipated in the older population of men with “age-related hypogonadism).^8,9^

Given this concern, these two products were approved with a boxed warning, FDA's strongest warning, stating that the prescriber should consider the patient's baseline cardiovascular risk and ensure blood pressure is adequately controlled, periodically monitor for hypertension or exacerbation of pre-existing hypertension, and re-evaluate whether the benefits of the drug outweigh its risks in patients who develop cardiovascular risk factors or cardiovascular disease on treatment. In addition, these products are contraindicated in men with hypogonadal conditions, such as “age-related hypogonadism,” that are not associated with structural or genetic etiologies. These are the only testosterone products to date with a contraindication for hypogonadal conditions that are not associated with structural or genetic etiologies, including “age-related hypogonadism.” This contraindication means that FDA has determined that the drugs' benefits never outweigh the risks for these conditions, given that their effectiveness has not been established for treatment of these conditions and the observed increases in blood pressure (which may not be readily detected in clinical practice) can increase the risk of MACE in these patients over time.

Because other testosterone products have not routinely undergone ambulatory blood pressure monitoring as part of their development programs, it is unclear at this time the extent to which the blood pressure findings with subcutaneous testosterone enanthate and oral testosterone undecanoate apply to other members of the testosterone drug class. Therefore, FDA has required the companies that own testosterone products to conduct an ambulatory blood pressure monitoring study with their testosterone drugs to determine whether those drugs also increase blood pressure. These required trials are ongoing. Until those results are available, we do not know whether these small but clinically relevant increases in blood pressure seen with these two newly approved products reflect a testosterone class effect or are specific to these testosterone formulations or their routes of administration.

What about new data that evaluate the benefits of testosterone use for “age-related hypogonadism”? The testosterone trials, published after the 2014 advisory committee meeting, were randomized double-blind placebo-controlled trials designed to address the key efficacy issues identified in the 2004 Institute of Medicine Report related to the lack of definitive evidence of benefit of testosterone therapy in older men. These trials assessed the effects of testosterone in older men on anemia, bone mineral density, cardiovascular status, cognition, physical function, sexual function, and vitality. For most of these measures, the publications reported no improvements with testosterone therapies, and for others, the results were of uncertain clinical benefit.^10–13^ In addition, these trials were not designed to definitively address the safety question of whether there is cardiovascular harm with use of testosterone for treatment of “age-related hypogonadism.”

In summary, although there still appears to be considerable use of testosterone for “age-related hypogonadism,” the labeling for testosterone therapies either states that the effectiveness and safety of such use have not been established or that such use is contraindicated. We recognize that recommendations and guidelines from professional societies on testosterone use for clinical conditions consistent with “age-related hypogonadism” do not necessarily align with the FDA-approved use of testosterone. The FDA approves a drug, including testosterone, for its intended use only when data submitted to FDA show benefits for such use demonstrated with substantial evidence from adequate and well-controlled trials and those benefits outweigh the known and potential risks. Once the FDA approves a drug, a health care provider may prescribe the drug for an unapproved use when the health care provider judges that it is medically appropriate for the patient. FDA does not regulate this off-label use, which is considered the practice of medicine. However, because most of the testosterone use is in patients with “age-related hypogonadism,” it is important for the public to know that the FDA is not aware of substantial evidence from adequate and well-controlled trials that establish benefits of testosterone in these men in the face of significant known and potential safety concerns with these drugs.

## Abbreviation Used

MACE: major adverse cardiac event

## Notes

^i^ Packages indicates boxes, bottles (including pump bottles), packets, jars, vials, pens, and tubes of testosterone product.

## References

[1] FDA briefing document for the joint meeting of the Bone, Reproductive, and Urologic Drugs Advisory Committee (BRUDAC) and the Drug Safety and Risk Management Advisory Committee (DSARMAC). Silver Spring, MD: FDA Advisory Committee. 2014

[2] Institute of Medicine. Testosterone and Aging: Clinical Research Directions. Washington: The National Academies Press. 2004 25009850

[3] Nguyen C, Hirsch M, Moeny D, Kaul S, Mohamoud M, Joffe HV. Testosterone and “Age-Related Hypogonadism.” N Engl J Med 2015;373:689–691 10.1056/NEJMp1506632 PMC8905399 26287846

[4] IQVIA Total Patient Tracker™. www.iqvia.com (accessed 1027, 2020)

[5] Syneos Health Research & Insights TreatmentAnswers™. www.syneoshealth.com (accessed 1126, 2019)

[6] IQVIA PharMetrics^®^ Plus. www.iqvia.com (accessed 1026, 2020)

[7] FDA Press Release: FDA approves new oral testosterone capsule for men with certain forms of hypogonadism. https://www.fda.gov/news-events/press-announcements/fda-approves-new-oral-testosterone-capsule-treatment-men-certain-forms-hypogonadism (accessed 1026, 2020)

[8] FDA Guidance for Industry: Assessment of Pressor Effects of Drugs, May 2018. https://www.fda.gov/media/113477/download (accessed 1026, 2020)

[9] Prospective Studies Collaboration. Age-specific relevance of usual blood pressure to vascular mortality: A meta-analysis of individual data for one million adults in 61 prospective studies. Lancet 2002;9349(360):1903–1913 10.1016/s0140-6736(02)11911-8 12493255

[10] Snyder PJ, Bhasin S, Cunningham GR, et al. Effects of testosterone treatment in older men. N Engl J Med 2016;374(7):611–624 26886521 10.1056/NEJMoa1506119 PMC5209754

[11] Snyder PJ, Koperdahl D, Stephens-Shields A, et al. Effect of testosterone treatment on volumetric bone density and strength in older men with low testosterone: a controlled clinical trial. JAMA Intern Med. 2017;177(4):471–479 28241231 10.1001/jamainternmed.2016.9539 PMC5433755

[12] Resnick S, Matsumoto A, Stephens-Shields J, et al. Testosterone treatment and cognitive function in older men with low testosterone and age-associated memory impairment. JAMA. 2017;317(7):717–727 28241356 10.1001/jama.2016.21044 PMC5433758

[13] Roy C, Snyder PJ, Stephens-Shields A, et al. Association of testosterone levels with anemia in older men: a controlled clinical trial. JAMA Intern Med. 2017;177(4):480–490 28241237 10.1001/jamainternmed.2016.9540 PMC5433757

